# HIV-1 Envelope gp41 Broadly Neutralizing Antibodies: Hurdles for Vaccine Development

**DOI:** 10.1371/journal.ppat.1004073

**Published:** 2014-05-22

**Authors:** Laurent Verkoczy, Garnett Kelsoe, Barton F. Haynes

**Affiliations:** 1 Duke Human Vaccine Institute, Duke University School of Medicine, Durham, North Carolina, United States of America; 2 Department of Medicine, Duke University School of Medicine, Durham, North Carolina, United States of America; 3 Department of Pathology, Duke University School of Medicine, Durham, North Carolina, United States of America; 4 Department of Immunology, Duke University School of Medicine, Durham, North Carolina, United States of America; Columbia University, United States of America

A primary correlate of protection for most effective viral vaccines is induction of antibodies with potent virus neutralization [1]. HIV-1 differs from other viruses for which successful vaccines have been made, because as a highly mutable, integrating retrovirus, it is resistant to both immune responses and antiretroviral therapy upon establishment of a latently infected CD4^+^ T-cell pool [2]. Thus, to prevent infection, an HIV-1 vaccine must induce protective immunity that is active during transmission [3]. Broadly neutralizing antibodies (bnAbs) are targeted to conserved regions of the HIV-1 envelope (Env) and neutralize a broad spectrum of HIV-1 quasi-species [4]. When passively infused in rhesus macaques [5]–[8] or transduced in humanized mice [9] preceding challenge, bnAbs robustly prevent infection, suggesting they can protect if present during transmission. However, bnAbs are made in a minority of HIV-1–infected individuals years after infection and cannot be elicited by current immunization regimens [3]. Thus, identifying impediments to bnAb induction to devise better immunization strategies is a central goal for HIV-1 vaccine development.

## Potential Structural and Immunological Roadblocks to BnAb Induction

The advent of high-throughput recombinant antibody technology has facilitated isolation of bnAbs with remarkable breadth and potency from HIV-1–infected individuals [10] and has re-invigorated structure-based vaccine design efforts [4]. We now have atomic-level descriptions of multiple bnAb epitopes and extensive knowledge of four general, vulnerable Env regions in which they cluster: the gp41 membrane proximal external region (MPER), the gp120 CD4-binding site (CD4bs), and two sites enriched for quaternary proteoglycan epitopes, one associated with the first/second hypervariable loops (V1/V2-glycan), the other around the third hypervariable loop (V3-glycan) [3], [4], [10]. Despite this progress, efforts to engineer immunogens capable of presenting neutralizing epitopes still fail to induce bnAbs [4], making it increasingly apparent that more traditional approaches, i.e., those aimed at overcoming limited bnAb epitope accessibility resulting from steric and conformational hindrances [3] or eliminating immunogenically dominant non-neutralizing epitopes [11], while important, cannot alone solve the HIV-1 bnAb induction problem.

Therefore, attention has instead shifted to the host for insight into why bnAbs are so difficult to induce. This re-focus is derived from observations that two gp41 bnAbs exhibited features suggesting immune tolerance mechanisms limited their induction [12], [13]. One of these features, in vitro poly/autoreactivity, has reasonable concordance with immune tolerance, as suggested by the observation that in vitro poly/autoreactivity of the normal human repertoire progressively wanes at developmental stages, coinciding with previously defined tolerance checkpoints [14] (reviewed in [15]). A second feature, extended antibody combining regions (HCDR3s), can also invoke counterselection of B-cells, either during peripheral development [16] or prior to B-cell receptor (BCR) expression [17], especially when as accentuated as in the V1/V2-glycan class of bnAbs [4], [15]. We now know from knock-in (KI) models of MPER-targeting bnAbs that such traits can invoke profound immune tolerance [18]–[22], and other recent studies have revealed this bnAb class may be modulated by additional contributory immunoregulatory factors: either genetic determinants like V_H_ allelic variation [23] and overlapping MHC class II–restricted CD4^+^ T_H_/bnAb epitopes [24] or environmental influences, such as shaping of the B-cell repertoire by incidental antigen exposure [25]. The isolation of over 100 bnAbs has not only confirmed the presence of long HCDR3s and/or poly/autoreactivity in most, but has revealed a third feature in all: unusually high somatic mutation levels that may also be associated with self-reactivity [26] and, if required by vaccination, cannot be achieved by existing immunization protocols [10], [26].

## Evidence for Tolerance Control of Self-Reactive BnAbs

4E10 and 2F5, HIV-1 Env gp41 bnAbs with adjacent, linear epitopes in the MPER and two of only three bnAbs to have been directly isolated from HIV-1–infected patients between 1993 and 2009, were identified to have traits associated with negative selection, i.e., long HCDR3s and poly/autoreactivity [12]. Based on this, we proposed that bnAb responses were impaired by immune tolerance [12], [13]. Recently, we and others have examined this hypothesis by following B-cell development in KI mice that express 4E10 or 2F5 V(D)J rearrangements [18]–[22]. These mice exhibited striking blockades in generating immature B-cells, a phenotype characteristic of central clonal deletion and similar to KI mice expressing BCRs with high affinities to known self-antigens [27]–[29]. Furthermore, residual 2F5 and 4E10 KI B-cells poorly express and signal through their BCRs [19]–[22], resembling unresponsive (anergic) B-cells [30]. Overall, these results indicate that the self-reactivity of the 2F5 and 4E10 bnAbs is sufficient to induce profound immune tolerance-mediated negative selection in vivo.

Recently, conserved vertebrate host antigens recognized by 2F5 and 4E10 have been identified: kynureninase and splicing factor 3b subunit-3 (SF3B3), respectively [31]. Interestingly, 2F5's bnAb epitope ELDKWA is identical to an alpha-helical motif in kynureninase [31], and B-cell escape clones from 2F5 KI mice selectively and stringently purge ELDKWA binding [20], [32]. In contrast, 4E10's bnAb epitope NWF(N/D)IT is not present in SF3B3, and relative to 2F5, 4E10 exhibits considerably more polyreactivity [31] with high avidity for lipids [13]. Thus, for certain bnAbs, like 2F5, viral mimicry may involve substantial sequence overlap between host and bnAb epitopes.

These studies [19]–[21], [31], [32] challenge the widely held immunologic notion that self- and viral epitope specificities are distinct, and they raise an intriguing question: to what extent does self-mimicry generally impact antiviral responses? Regarding bnAb responses specifically, a key question for HIV-1 vaccine development will be whether immune tolerance limits induction of all MPER^+^ bnAbs and to what extent it limits bnAbs targeting other Env regions. The recent identification of 10E8, a potent MPER^+^ bnAb lacking in vitro polyreactivity [33], argues against the former question, but only if standard autoimmune assays for poly/autoreactivity reflect bona fide physiological self-reactivity. Indeed, since work using protein arrays has identified 10E8 specificity for select host-antigens (Kelsoe G, Haynes BF, unpublished results, manuscript in preparation), generation of 10E8 KI mice will be needed to resolve this issue. Also noteworthy is that bnAb b12, originally reported to be poly/autoreactive [13], lacks tolerizing self-reactivity in KI mice [34]. Although b12's origin from a phage library limits the physiological relevance of this example, it nevertheless reinforces the importance of using bnAb KI models to confirm in vivo effects of poly/autoreactivity measurements.

A key, related issue regarding how tolerizing self-reactivity impairs bnAb induction is when it is acquired or lost in various bnAb lineages. Since KI mice carrying reverted (unmutated ancestor [UA]) 2F5 BCRs undergo profound central deletion (Verkoczy L, Haynes BF, unpublished results, manuscript in preparation), bnAbs like 2F5 likely have self-reactivity encoded in their primary VDJ rearrangement (or, at least, early during affinity maturation, given the caveat in predicting its inferred HCDR3 using best-probability estimates), while others like CH103 and 4E10, whose UA BCRs lack bnAb and self-epitope specificity in vitro (Haynes BF, unpublished results, and [35], [36]) may be tolerized in the periphery. Even bnAbs, like VRC01 and 10E8, that may lack tolerizing self-reactivity and bear considerably more mutations than polyreactive bnAbs to similar epitopes could themselves be products of tolerized bnAb lineages (see below). Thus, definitively evaluating the extent to which self-reactivity limits bnAb lineages will likely require assessing in vivo tolerizing effects of gene-targeted V(D)J specificities of not only bnAbs but also their lineage ancestors. In this regard, higher-throughput KI methodologies, based on RAG blastocyst complementation [37], could significantly accelerate such analysis.

## BnAbs Arise after Extensive Somatic Mutation and Virus Evolution during HIV-1 Infection

A hallmark of B-cells is their ability to interact with exogenous antigen and to produce specific Abs with high affinity. This process, affinity maturation (AM), occurs in germinal centers by two linked mechanisms, IgV region somatic hypermutation and affinity-dependent selection. In secondary responses to conventional antigen, somatic mutation levels are normally restricted, because excess mutation decreases affinity and cell survival [38]. In contrast, bnAbs accumulate high (15%–48%) mutation frequencies [4], which may be required for neutralizing activity by promoting structural flexibility in bnAb V(D)J framework regions [39]. That all bnAbs identified thus far originate from subjects infected with HIV-1 for 2–4 years suggests that these remarkable mutation frequencies are a product of highly convoluted, yet-to-be understood AM pathways [26], but how and why this occurs has been elusive.

Studies examining the in vivo evolution of clonal bnAb lineages during chronic HIV-1 infection [35], [40] now provide insight into how selection pressures by Env sequence diversification impacts this process. In a study by Liao et al., the entire evolutionary footprint of CH103 (a CD4bs^+^ bnAb derived from a donor that seroconverted after ∼2.5 years) and its virus targets were molecularly elucidated [35]. Interaction of this bnAb lineage UA BCR with the transmitted founder virus Env resulted in intense co-evolution by successive viral escape mutants and mutant BCR intermediates, culminating in viral diversification focused to the restricted CH103 epitope, immediately preceding final acquisition of neutralizing breadth.

These studies, and recent findings that most experimentally reverted bnAb UAs lack neutralizing epitope affinity [26], [34], [41], provide one plausible explanation for why bnAbs accumulate so much mutation: naïve BCRs need to be extensively modified to meet unusually demanding structural requirements for acquiring breath and/or potency, i.e., significant affinity to bnAb epitopes. Could alternative explanations account for this degree of mutation in some bnAbs? That bnAbs with in vitro poly/autoreactivity tend to be less mutated relative to non-poly/autoreactive bnAbs for similar epitopes [4], [15] raises the intriguing possibility that tolerance and extensive AM, in bnAbs whose epitopes have structural overlap with self-antigens, are causally linked. Two mechanisms by which tolerizing self-reactivity of bnAbs could drive unusually high mutation rates have been proposed [26], [31], [32]. First, negative selection during early development may forbid unmutated BCRs that bind self-mimicking bnAb epitopes to participate in conventional AM, thus creating “holes in the repertoire” that necessitate recruitment of weakly crossreactive, previously mutated B-cell clones to achieve bnAb specificity via AM. The second non-exclusive possibility is that there is close, but not complete, structural overlap between self- and bnAb epitopes. Thus, already-mutated B-cells with neutralizing specificity attempt to escape anergy via selection for mutations at critical V(D)J residues that remove self-reactivity but maintain bnAb epitope affinity, a requirement that may take multiple rounds of mutation and selection to accomplish.

## Developing Novel BnAb-Based HIV-1 Vaccine Strategies

**Figure 1:**
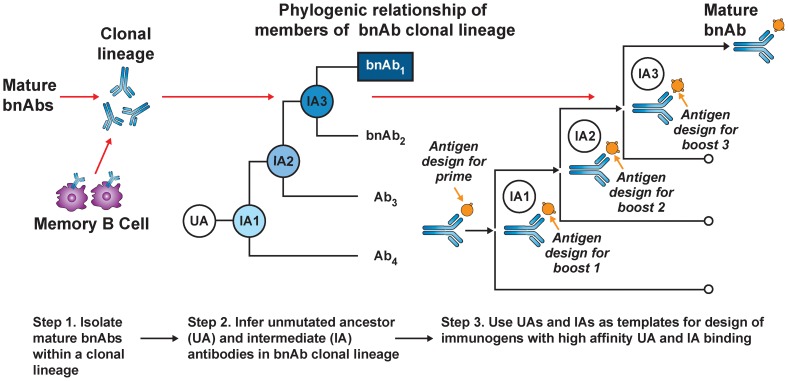
B-cell lineage-based approach to vaccine design. Mature bnAbs can be isolated from HIV-1–infected donors using modern methods such as memory B-cell culture or sorting of antigen-specific B-cells. Based on the known bnAb sequence, next generation sequencing can be used to find numerous clonal relatives of the mature bnAb. If appropriate longitudinal samples are available, it is possible to infer the full antibody lineage, including the UA and IAs. The expressed UA and IA sequences can then be used as templates for the design of HIV-1 immunogens with high-affinity binding. As the antibody lineage is known to evolve in response to viral evolution, it may be possible to design sequential immunogens with high-affinity binding for the UA and IA, thus guiding the antibody response toward the mature antibody with broad neutralizing activity (with permission from [4]).

Current HIV-1 vaccination strategies induce antibody mutation profiles (∼4%–5%) similar to autologous neutralizing antibodies in acute HIV infection and most human antibodies produced in 2° responses to infection [10]. Thus, the apparent requirement for polyreactivity and/or extensive mutation for generating bnAb function [39], [42], [43] comes at an expensive cost: creation of disfavored and/or complex AM pathways that existing vaccine approaches cannot recapitulate. Recently, a B-cell lineage immunogen design approach has been proposed to overcome this (Figure 1) [26]. This strategy builds on two assumptions: (1) the best immunogens bind naïve BCRs with the highest affinity, and (2) serial immunization with distinct immunogens can recreate Env-guided AM pathways that generate bnAbs during infection. The general approach involves priming with an immunogen that binds a given bnAb lineage's inferred UA BCR (to initiate B-cell responses), followed by serial boosting with immunogens optimized to bind inferred intermediate ancestor (IA) BCRs. Perhaps the most straightforward and physiological use of this strategy for development of HIV-1 vaccine candidates would involve sequential use of Env proteins identified from serial isolates of known bnAb lineages to drive evolutionary intermediates [35]. Variations of this approach have also been recently proposed, involving in vitro selection and/or mutational methodologies to synthetically engineer immunogens that bind both UA and mature BCRs from bnAb lineages to common epitopes [44], [45], and more generally, analogous vaccine strategies against other pathogens have also recently been described [46].

## Conclusions

Although our studies suggest host mimicry by HIV-1 Env gp41 neutralization epitopes limits bnAb induction [19]–[21], [31], [32], they also indicate such epitopes can be used in vaccine regimens designed to successfully target anergic bnAb-specific B-cells [32]. Furthermore, poly/autoreactivity is part of normal antibody responses [43], and evidence from passive protection or in vitro pathogenicity assays thus far does not suggest adverse effects would result from inducing bnAbs with tolerizing self-reactivity [4]. However, the requirement for high mutation levels [39] may be the most daunting problem facing HIV-1 vaccine development and may necessitate identifying and exploiting lineages requiring relatively fewer mutations to acquire bnAb specificity. Finally, since single bnAbs readily select HIV-1 escape mutants [4], [35], but certain combinations of individual bnAbs targeting distinct Env regions (for example, V1/V2-glycan and CD4bs-specific bnAbs) confer near-complete breadth [10], a successful vaccine will also need to incorporate approaches capable of inducing multiple types of bnAbs.
